# Robust Gene-Gene Interaction Analysis in Genome Wide Association Studies

**DOI:** 10.1371/journal.pone.0135016

**Published:** 2015-08-12

**Authors:** Yongkang Kim, Taesung Park

**Affiliations:** 1 Department of Statistics, Seoul National University, Seoul, South Korea; 2 Interdisciplinary Program in Bioinformatics, Seoul National University, Seoul, 151–741, South Korea; Indiana University Bloomington, UNITED STATES

## Abstract

Genome-wide association studies (GWAS) have successfully discovered hundreds of associations between genetic variants and complex traits. Most GWAS have focused on the identification of single variants. It has been shown that most of the variants that were discovered by GWAS could only partially explain disease heritability. The explanation for this missing heritability is generally believed to be gene-gene (GG) or gene-environment (GE) interactions and other structural variants. Generalized multifactor dimensionality reduction (GMDR) has been proven to be reasonably powerful in detecting GG and GE interactions; however, its performance has been found to decline when outlying quantitative traits are present. This paper proposes a robust GMDR estimation method (based on the L-estimator and M-estimator estimation methods) in an attempt to reduce the effects caused by outlying traits. A comparison of robust GMDR with the original MDR based on simulation studies showed the former method to outperform the latter. The performance of robust GMDR is illustrated through a real GWA example consisting of 8,577 samples from the Korean population using the Homeostasis Model Assessment of Insulin Resistance (HOMA-IR) level as a phenotype. Robust GMDR identified the KCNH1 gene to have strong interaction effects with other genes on the function of insulin secretion.

## Introduction

A genome-wide association (GWA) study has become a common approach for testing the association between a single nucleotide polymorphism (SNP) and a complex trait of interest [1]. There have been many successful results from genome-wide association studies (GWAS). However, SNPs that were identified by GWAS have been shown to explain only a small fraction of disease etiology, because the relatedness between complex diseases and multiple genes and/or their interactions are ignored. For this reason, the analysis of gene-gene (GG) and gene-environment (GE) interactions have been emphasized as a new alternative for understanding the etiology of common complex traits. However, GG and GE interactions are hard to detect and characterize by using traditional parametric statistical methods, for the following reasons [2]. First, high dimensional genetic data may be of a sparse nature. The issue of data sparseness can be addressed by using exponentially large sample sizes when parametric statistical methods are used for determining GG and GE interactions. Second, detecting GG and GE interactions using traditional procedures may lead to an increase in type II errors and a decrease in power. As a result, detecting GG and GE interactions has been a well-known challenge in statistics and data mining areas [3].

Ritchie et al. proposed a Multifactor Dimensionality Reduction (MDR) method for detecting GG interaction [4]. This method is not affected by data sparsity because it allows multilocus genotype combinations with very few or no data points. However, the original MDR method can only be used in association studies for qualitative traits. The MDR method was extended to more general types of traits, including quantitative traits, by Lou et al., who proposed the Generalized Multifactor Dimensionality Reduction (GMDR) method [5]. This method is quite flexible in handling various types of traits and is even able to account for individual covariates.

Since the initial introduction of the original MDR and GMDR methods, numerous extensions of MDR have been developed. Calle et al. proposed model-based MDR (MB-MDR) for the parametric extension of the MDR method [6]. Recently, Quantitative MDR (QMDR) was proposed for handling quantitative traits [7].

These MDR methods have brought many remarkable successes in GG interaction analysis. However, the effects of outlying trait observations on the results of MDR methods have not been studied. Outlying trait observations are those traits that diverge from the global distribution of traits. Bennett et al., Most et al., and Yang et al. discussed the treatment of outliers in GWAS and proposed simple approaches, such as removing outlying observations and transforming traits, including log-transformation or inverse-normalization [8, 9, 10]. The R-package QCGWAS includes a process for detecting outliers by skewness or kurtosis. However, these QC methods are very sensitive to the underlying distributional assumption and may lead to the wrong conclusions when the distributional assumption is not met. In this paper, we first demonstrate the effects of outlying trait observations on GMDR analysis. Next, we propose robust GMDR for reducing the effects caused by outlying traits. Robust GMDR uses robust estimation based on L-estimator and M-estimator. Simulation studies were used to compare the robust GMDR developed in this work to GMDR, and was shown to outperform GMDR. The performance of robust GMDR is illustrated through a real GWA example consisting of 8,577 samples from the Korean population.

## Materials and Methods

### KARE data

The Korea Association Resource (KARE) is a project for gathering large-scale GWA analyses in the Gyeonggi Province of South Korea [11]. Two community cohorts participated in the KARE project: the Ansung and Ansan cohorts. The number of participants in the Ansung cohort is 5,018 and that in Ansan cohort is 5,020. The age of the participants were between 40 and 69, and more than 260 traits have been extensively examined through epidemiological surveys, physical examinations, and laboratory tests. In this paper, the Homeostasis Model Assessment of Insulin Resistance (HOMA-IR) levels are used, which are widely employed to estimate insulin resistance. As HOMA-IR has a nonnegative skewness distribution, a gamma distribution is commonly assumed [12]. DNA samples were genotyped on the Affymetrix Genome-Wide Human SNP array 5.0, which is able to genotype 500,568 SNPs. The quality control processes are well described in a study by Cho et al. [13]. For the purposes of our work, samples without Body Mass Index (BMI) or HOMA-IR scores were disregarded. After removing the samples with the missing phenotypes, a total of 8,577 individuals and 327,872 SNPs remained in the study.

### Review of MDR method

The evaluation of genetic effects using the MDR method requires several steps. The first step involves selecting genetic factors according to their effects on the phenotype. If GG interactions of the *n*th order are required, *n* SNPs are selected and a 3^*n*^ contingency table is created. Each cell of the contingency table contains the number of cases and controls with the same genetic factor combinations. The second step calculates the ratio of the number of cases to the number of controls in each cell, and uses this ratio to determine whether a cell is “high risk” or “low risk”. If the ratio of case to control in a cell exceeds the threshold (which is commonly specified as 1), then those cells are labeled “high risk”; otherwise they are labeled “low risk”. This process enables each cell to be classified as either “high risk” or “low risk”. In the third step, the ability of selected SNP sets to perform these classifications based on the case / control ratio are evaluated through accuracy and balanced accuracy (BA) measures, where BA is a measure for the prediction ability of the model and is defined as follows:

$$BA=\frac{1}{2}(sensitivity+specificity)$$ (1)

The larger the BA produced by the model, the more accurate the model’s prediction ability [14]. These measures are computed from the 2×2 confusion table in which the first variable represents the case / control ratio and the second variable high risk and low risk. Then, the sensitivity is defined as the ratio of the number of cases to the number of high risk samples and the specificity is defined as the ratio of the number of controls to low risk samples. All these steps would be exhaustively performed for all SNP combinations.

The problem of over-fitting is usually avoided by performing a 10-fold cross-validation. It is also possible to use the cross-validation consistency (CVC) as a measure for selecting SNP combinations. This selection is based on the number of times the same SNP combination is selected as best out of 10 cross-validated samples. Alternatively, the SNP combination with the largest CVC measure can also be selected as the best SNP combination. BA and CVC measures have been widely used in MDR analysis

### Review of GMDR method

Although MDR has been used extensively in GG interaction analysis, it has several limitations. First, the MDR method is only capable of handling qualitative traits such as case and control data, because the method classifies each cell by using the case / control ratio. Second, it is not possible for MDR to adjust for other individual covariates such as environmental factors. Therefore, using MDR to analyze traits that are affected by environmental factors may produce biased results. In an attempt to overcome these limitations, Lou et al. proposed the Generalized Multifactor Dimensionality Reduction (GMDR) method [5], each step of which is very similar to those of the original MDR method. However, instead of using the counts of individuals, the GMDR method uses a residual-based score.

Let *y*_*i*_ denote the phenotype of individual *i*, which can be either quantitative or qualitative. Let *E*(*y*_*i*_) = *μ*_*i*_. Consider the following generalized linear model (GLM):

$$l(\mu_{i})=\alpha +x_{i}^{T}\beta +z_{i}^{T}\gamma$$ (2)

where *l*(*μ*_*i*_) is the link function, α is the intercept, and *x*_*i*_ is a vector that expresses possible genotype combinations of interest. *z*_*i*_ is a vector representing environmental factors, and β and γ are coefficient vectors.

The first step when using GMDR is to fit the null model only with environmental factors under the null hypothesis, i.e., β = 0. The second step is to classify each SNP combination using the residuals from the null model. For each cell of the contingency table, the sum of the residuals is computed. Cells with a positive residual sum are classified as “high risk”; otherwise, they are classified as “low risk”. Subsequent GMDR cross-validation steps are performed similar to those of MDR.

It is noteworthy that, in this GMDR procedure, the distinction between high and low classification is based on the sum of the residuals. Thus, a few outlying observations may have a large impact on the performance of GMDR, which provided the motivation for considering robust GMDR. Although GLM can handle both quantitative and qualitative traits, our efforts were focused on a quantitative trait with outlying observations. Thus, an identity link function was used with the supposition that y_i_ has an independent normal distribution with equal variance.

### Proposed Robust GMDR method

The effect of outlying observations can be reduced by considering robust scores such as an L-estimator and an M-estimator type score. The L-estimator is a linear combination of order statistics that have to be measured. A well-known example of the L-estimator is least trimmed square (LTS) [15]. LTS regression is formulated as follows.

$$\arg\;\min_{\beta }\sum_{i=1}^{k}|\gamma_{\left(i\right)}(\beta ){|}^{2},\;where\;\gamma_{i}(\beta )=(y_{i}-f(x_{i},\beta )),\;i=1,\ldots ,\text{n}.$$ (3)

Here, *k* is the number of samples that is actually used in the regression and n is the total sample size. *γ*_*i*_(*β*) is the *i*th residual for a fixed β that is ordered according to the absolute value of the residuals. Bickel and Lehmann considered trimmed expectations to be the only ones which are both robust and whose estimators have guaranteed high efficiency [16]. LTS is known to outperform other least square estimators. Using LTS regression, our L-estimator GMDR uses a studentized trimmed residual as a score for GMDR, where the trimmed studentized residual is defined as follows:

$${\dot{\gamma }}_{i}(\beta )=\{\begin{array}{c} \frac{y_{i}-f(x_{i},\beta )}{\sqrt{\mathrm{var}(\hat{y_{i}-f(x_{i},\beta )}})}\;if\;\left|\frac{y_{i}-f(x_{i},\beta )}{\sqrt{\mathrm{var}(\hat{y_{i}-f(x_{i},\beta )}})}\right|\leq T\\ \;0\;if\;\left|\frac{y_{i}-f(x_{i},\beta )}{\sqrt{\mathrm{var}(\hat{y_{i}-f(x_{i},\beta )}})}\right|>T \end{array}$$ (4)

where T is the threshold value to determine whether the *i*th sample should be used, and it is possible to adjust T to determine the extent to which outliers would have to be trimmed.

The second robust GMDR uses an M-estimator type score, which is derived as the minima of the sums of the functions of the data. Least-squares estimators and many maximum-likelihood estimators are examples of M-estimators. The M-estimator was first derived for the purpose of introducing it into robust regression by Huber [17]. Tukey subsequently proposed a biweight function, which is a type of M-estimator for robust regression. The M-estimator using Tukey’s biweight function is defined as follows.

$$\rho (\gamma_{i}(\beta ))=\{\begin{array}{c} \frac{1}{6}{\left[1-{\left(1-\gamma_{i}(\beta )\right)}^{2}\right)}^{3}]\;if\;|\gamma_{i}(\beta )|\leq 1\\ \;\frac{1}{6}\;if\;|\gamma_{i}(\beta )|>1 \end{array},$$ (5)

$$\arg\;\min_{\beta }\sum_{i=1}^{k}\rho (\gamma_{i}(\beta ),\;where\;\gamma_{i}(\beta )=(y_{i}-f(x_{i},\beta )),\;i=1,\ldots ,\text{n}.$$ (6)

Solving the M-estimator requires differentiation of the ρ function, which usually requires selection of an appropriate shape for the biweight function.

Using the biweight function, our M-estimator GMDR defines the threshold residual score as:

$${\dot{\gamma }}_{i}(\beta )=\{\begin{array}{c} \frac{y_{i}-f(x_{i},\beta )}{\sqrt{\mathrm{var}(\hat{y_{i}-f(x_{i},\beta )}})}\;if\;\left|\frac{y_{i}-f(x_{i},\beta )}{\sqrt{\mathrm{var}(\hat{y_{i}-f(x_{i},\beta )}})}\right|\leq T\\ T\cdot sign(y_{i}-f(x_{i},\beta ))\;if\;\left|\frac{y_{i}-f(x_{i},\beta )}{\sqrt{\mathrm{var}(\hat{y_{i}-f(x_{i},\beta )}})}\right|>T \end{array}$$ (7)

In this formula, the threshold T is used to shrink all residuals exceeding T. As this function does not require differentiation; the biweight function is modified by simply reducing the weight caused by extreme loss.

## Results

### Simulation study

First, we simulated the phenotypes from the Normal distribution. In this case, samples were generated by using the following model.

$$y=envir_{1}+\beta_{1}SNP_{1}+\beta_{2}SNP_{2}+\beta_{1\times 2}SNP_{1}SNP_{2}+\varepsilon ,$$ (8)

$$envir_{1}\sim N(0,1),SNP_{1},\ldots ,SNP_{p}\sim Bin(2,MAF),\varepsilon \sim N(0,1).$$

The effects of MAFs were checked by considering two MAF values: 0.1 and 0.3. Next, outlying samples were generated by using the following model

y~N(0,400) (9)

$$SNP_{1},\ldots ,SNP_{p}\sim Bin(2,MAF).$$

A series of designs were created by using different settings for the purpose of data simulation, and the settings are summarized in Table 1. The first three columns of Table 1 denote the effect of the size of SNP1, SNP2, and the interaction in pure samples, respectively. The fourth column lists the mixed proportion, i.e., the proportion of outlying samples as generated by Model (9). Larger mixed proportions indicate that the particular simulation design generates more outlying observations. The fifth and sixth columns of Table 1 denote the heritability of each simulation setting when MAF = 0.1 and 0.3, respectively. The detailed equations for calculating the heritability are described in the Appendix.

**Table 1:** Models of simulation with Normal distribution.

	SNP1	SNP2	interaction	mixed proportion	Heritability (MAF = 0.1)	Heritability (MAF = 0.3)
Design1	**0.2**	**0.2**	**0.18**	**0.067**	**0.010**	**0.041**
Design2	**0.2**	**0.2**	**0.18**	**0.033**	**0.010**	**0.041**
Design3	**0.4**	**0.4**	**-0.3**	**0.067**	**0.022**	**0.027**
Design4	**0.4**	**0.4**	**-0.3**	**0.033**	**0.022**	**0.027**
Design5	**0.25**	**0.25**	**0.15**	**0.067**	**0.014**	**0.048**
Design6	**0.25**	**0.25**	**0.15**	**0.033**	**0.014**	**0.048**
Design7	**0.2**	**0.2**	**0.18**	**0**	**0.010**	**0.041**

The first to third columns denote the effect sizes of SNP1, SNP2 and interaction in pure samples respectively. The fourth column of Table 1 indicates mixed proportion. The fifth and sixth columns of Table 1 indicates heritability and skewness respectively.

Second, we performed simulation studies for the skewed distribution. The samples were generated from the gamma distribution given in (10). The MAF of SNPs was set to 0.3. The outlying observations were generated by the shifted Gamma distribution in (11). As the Gamma distribution has a long tail to the right, outlying observations were only generated for the right-hand side of the distribution. A series of designs are summarized in Table 2

y~Gamma(α,β) (10)

y~0.1+Gamma(α,β) (11)

α=(envir1+β1SNP+1β2SNP+2β3SNPS1NP)22,β=1/(envir1+β1SNP+1β2SNP+2β3SNPS1NP)2,

$$envir_{1}\sim N(0,1),SNP_{1},\ldots ,SNP_{p}\sim Bin(2,0.3)`.$$

**Table 2:** Models of simulation with Gamma distribution.

	SNP1	SNP2	interaction	mixed proportion	Heritability	Skewness
**Design 1**	**0.6**	**0.6**	**0.3**	**0.067**	**0.117**	**0.770**
**Design 2**	**0.6**	**0.6**	**0.3**	**0.033**	**0.117**	**0.8**
**Design 3**	**1.2**	**0.4**	**0.3**	**0.067**	**0.197**	**0.617**
**Design 4**	**1.2**	**0.4**	**0.3**	**0.033**	**0.197**	**0.587**
**Design 5**	**0.8**	**-0.4**	**-0.2**	**0.067**	**0.074**	**0.965**
**Design 6**	**0.8**	**-0.4**	**-0.2**	**0.033**	**0.074**	**0.801**
**Design 7**	**0.6**	**0.6**	**0.3**	**0**	**0.117**	**0.602**

The first column shows MAF of each design. Second to fourth columns denote the effect sizes of SNP1, SNP2 and interaction in pure samples respectively. The fifth and sixth columns of Table indicates heritability and skewness respectively.

For these Normal and Gamma distributions, we considered two sample sizes of 1,000 and 3,000 to evaluate the effect of sample size, and assumed the number of SNPs to be 100 and 1000. Ordinary GMDR, L-estimator-based GMDR, and M-estimator-based GMDR were compared in terms of their power of detecting a true SNP pair. We used Eq (12) for calculating the power, where N refers to the total number of iterations.

$$\frac{1}{N}\sum_{i=1}^{N}I(\text{set}\;\{SNP_{1},SNP_{2}\}\;\text{is selected in}\;i\text{th}\;\text{iteration}).$$ (12)

The number of iterations was 1,000 when the number of SNPs is 100, while it was 100 when the number of SNPs is 1000. The best SNP pair was selected by using the average CVC through a 10-fold cross validation.

We also computed the false detection rate (FDER) [18], which was done by assuming that a targeted non-causal SNP pair, say *SNP*_1_ and *SNP*_2_, exists. To detect this pair, we simulated 500,000 null datasets by permuting the response variables. Because there were at most 100 SNPs in each dataset, the randomly selected rate of each SNP pair is at least 1/499,500. We counted how many times the pair of *SNP*_1_ and *SNP*_2_ was selected as the best model. We found all three methods selected the pair of *SNP*_1_ and *SNP*_2_ at most twice when the number of SNPs is 1000, which shows that the three methods have a very low FDER.

**Fig 1:**
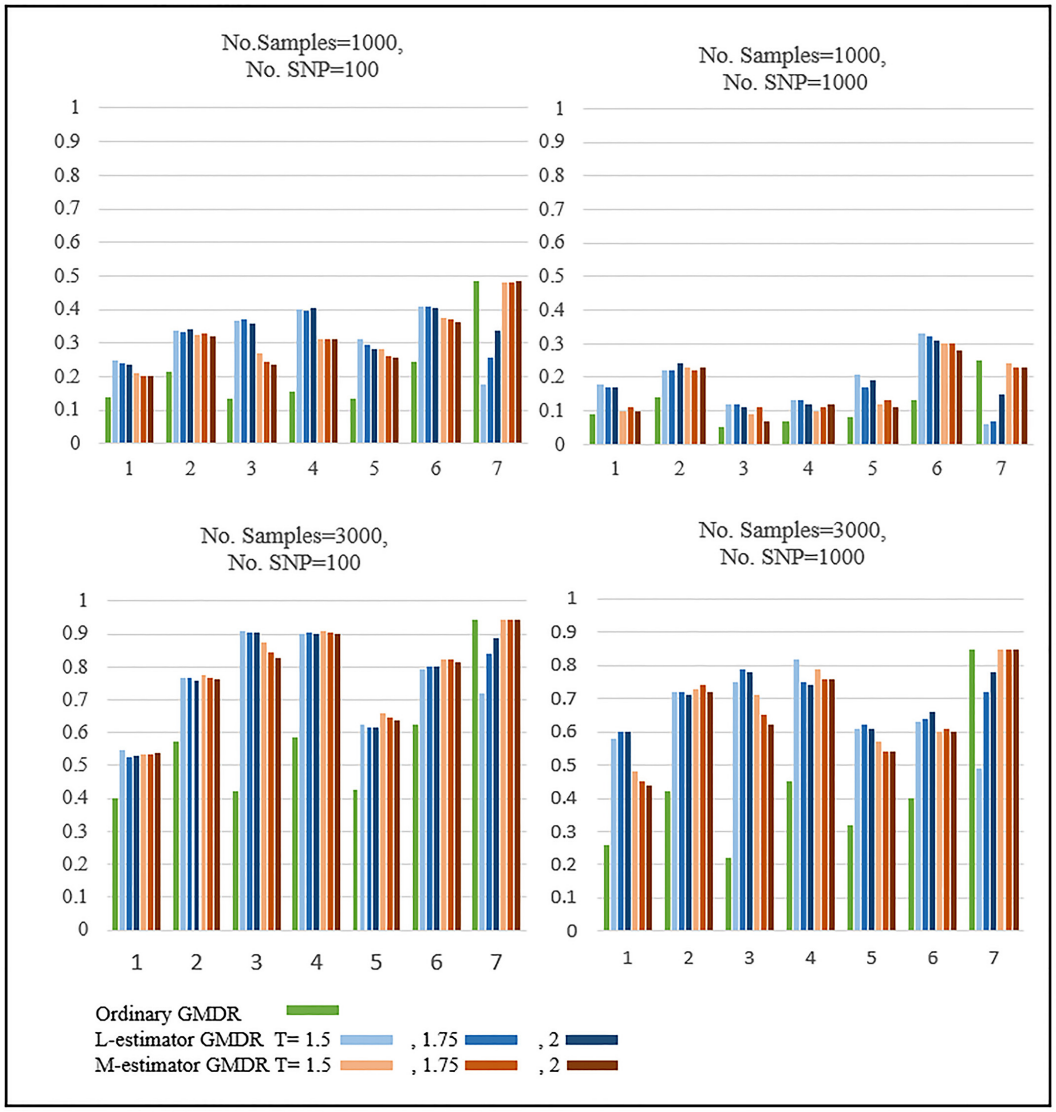
Results of Simulation: Normal distribution with MAF 0.3. The upper and lower figures show the results when the number of samples is equal to 1,000 and 3,000, respectively. These four figures almost show the same patterns, but the simulation power is different.

Fig 1 shows the results of the power comparison when SNPs are generated by Bin(2,0.3) and the phenotypes are obtained by Normal distributions. Designs 1 to 6 show the results when outlying observations exist and Design 7 when outlying observations do not exist. For Design 7, the ordinary GMDR and M-estimator GMDR outperform L-estimator GMDR. As L-estimator GMDR uses a smaller number of samples than other methods, the power of L-estimator GMDR is less than that of the others. For all the other designs, the performance of L-estimator GMDR and M-estimator GMDR exceeds that of ordinary GMDR.

**Fig 2:**
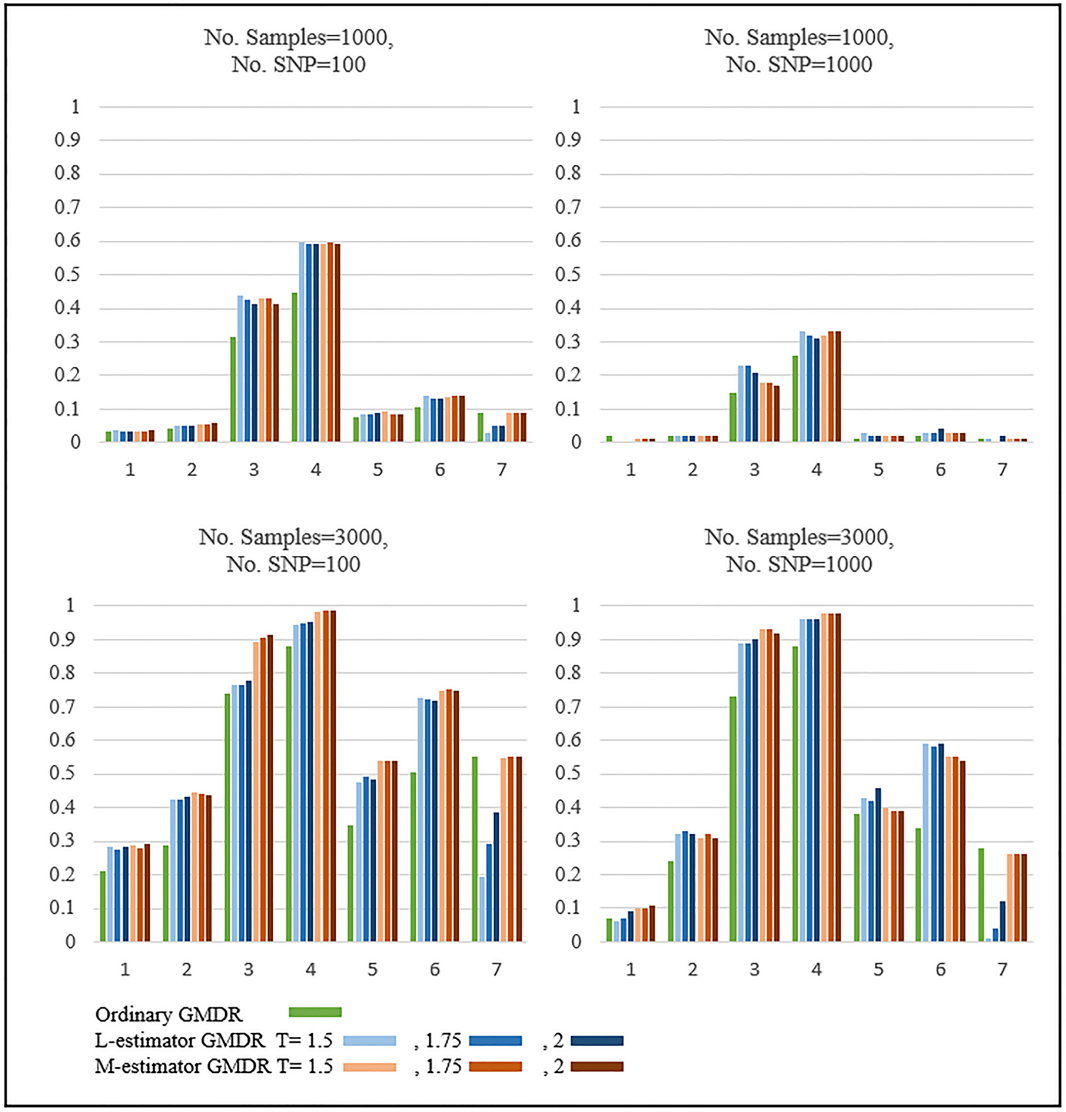
Results of Simulation: Normal distribution with MAF 0.1. The upper figures show the results when the number of samples is equal to 1000 and the lower figures show the results when the number of samples equals 3,000. These four figures almost show the same patterns, but the simulation power is different.

Fig 2 shows the results of the power comparison when SNPs are generated by Bin(2,0.1) and the phenotypes are obtained by Normal distributions. The patterns are quite similar to those in Fig 1. For Designs 1 to 6, when outlying observations exist, the two robust GMDRs were found to outperform ordinary GMDR. For Design 7, when outlying observations do not exist, the performance of ordinary GMDR and M-estimator GMDR exceeds that of L-estimator GMDR.

**Fig 3:**
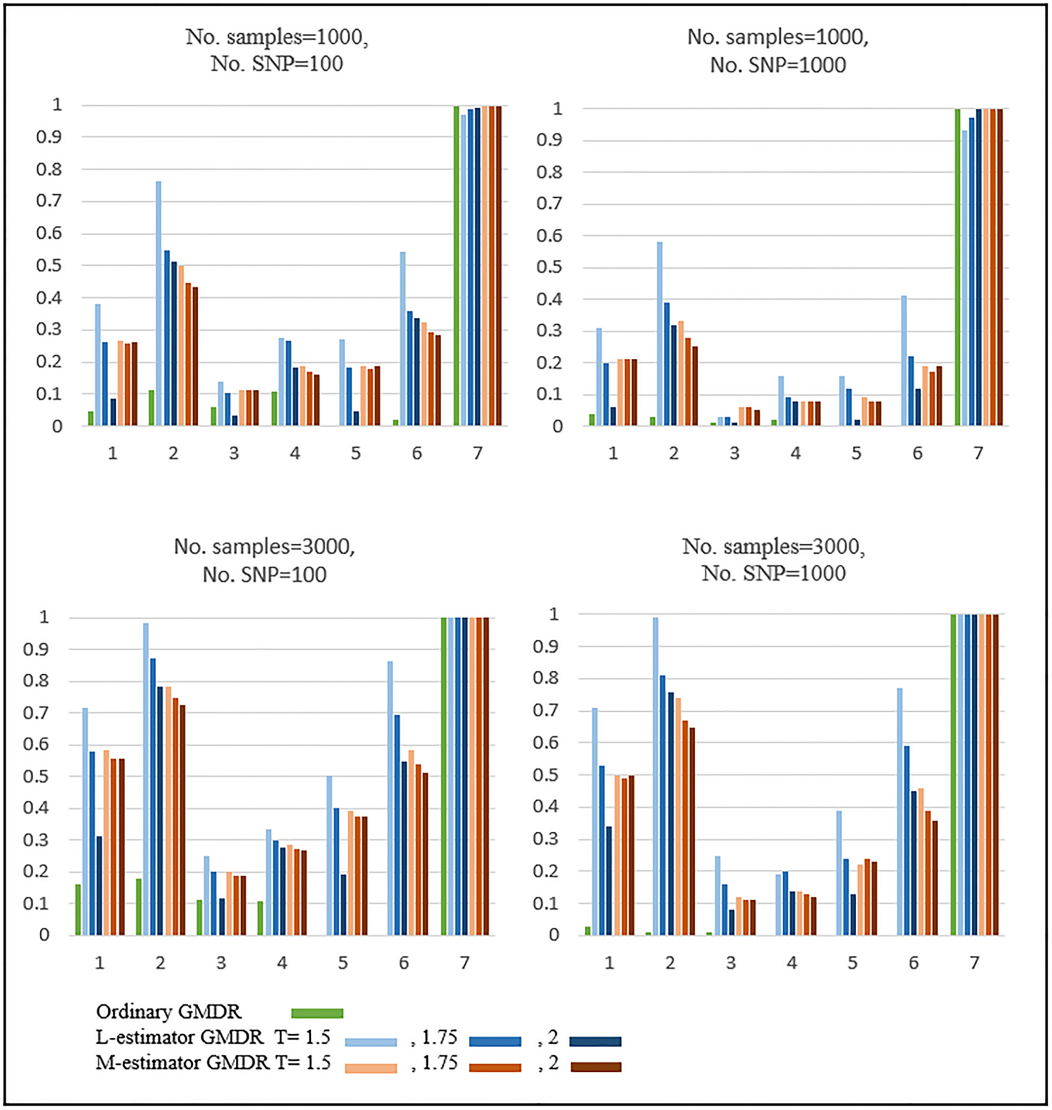
Results of Simulation: Gamma distribution with MAF 0.3. The upper figures show the results when the number of samples is equal to 1000 and the lower figures show the results when the number of samples equals 3,000. These four figures almost show the same patterns, but the simulation power is different.

Fig 3 shows the results of the power comparison when SNPs are generated by Bin(2,0.3) and the phenotypes are obtained by Gamma distributions. The residuals were calculated by generalized linear models under the Gamma distributional assumption. For Designs 1 to 6, when outlying observations exist, the two robust GMDRs were found to outperform ordinary GMDR to a large extent. In particular, L-estimator GMDR performed better than M-estimator GMDR. Note that the performance of L-estimator is highly dependent on the threshold values, whereas this is not the case for M-estimator. For Design 7, when outlying observations do not exist, all GMDRs perform similarly, probably due to the large SNP size effect.

**Fig 4:**
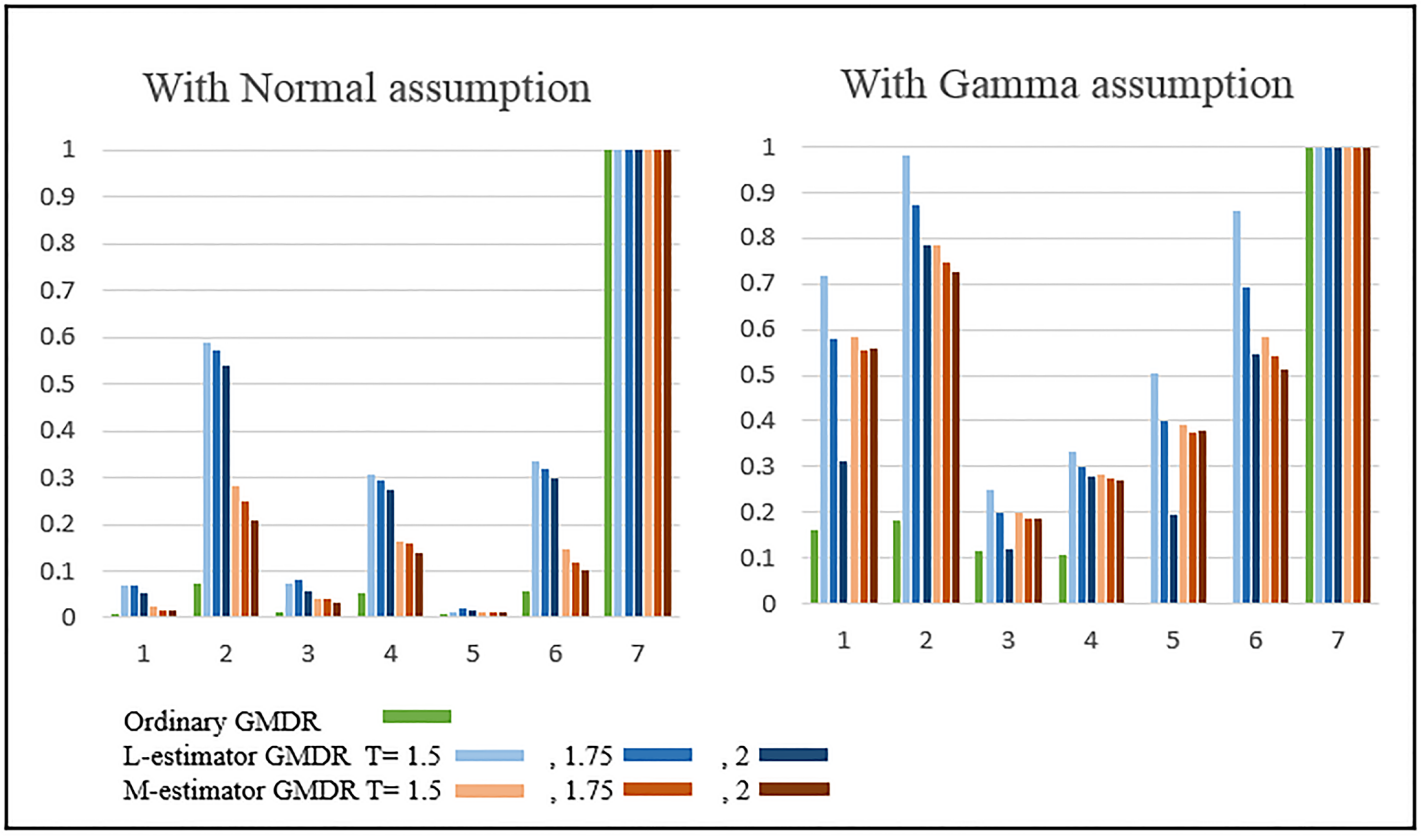
Results of Simulation: Power comparison between the residuals calculated under the Normal assumption and under the Gamma assumption. The residuals were calculated by generalized linear models under the Normal distributional assumption (left panel) and under the Gamma distributional assumption (right panel).

Fig 4 shows the results of the power comparison when SNPs are generated by Bin(2,0.3) and the phenotypes obtained by Gamma distributions. The residuals were calculated by generalized linear models under the Normal distributional assumption (left panel) and under the Gamma distributional assumption (right panel). In general, the use of GMDR in combination with the correct distributional assumption is much more powerful than with an incorrect distributional assumption. For Designs 1 to 6, when outlying observations exist, two robust GMDRs perform much better than ordinary GMDR. In particular, L-estimator GMDR outperforms M-estimator GMDR.

The effect of threshold values were investigated by using the robust GMDR methods with various threshold values, i.e., T = 1.5, 1.75, and 2. As shown in our simulation results in Figs 1, 2, 3 and 4, the dependence of L-estimator GMDR on T is much stronger than that of M-estimator GMDR. When the distribution of phenotypes is non-skewed (e.g., a Normal distribution), the effect of T is not large. On the other hand, when the distribution of phenotypes is skewed (e.g., a Gamma distribution), the effect of T is quite large. Although our simulation setting is limited, T = 1.5 consistently performed better than or equal to other threshold values in the presence of outlying observations. Thus, for practical applications, we recommend using T = 1.5.

### Real data analysis

**Fig 5:**
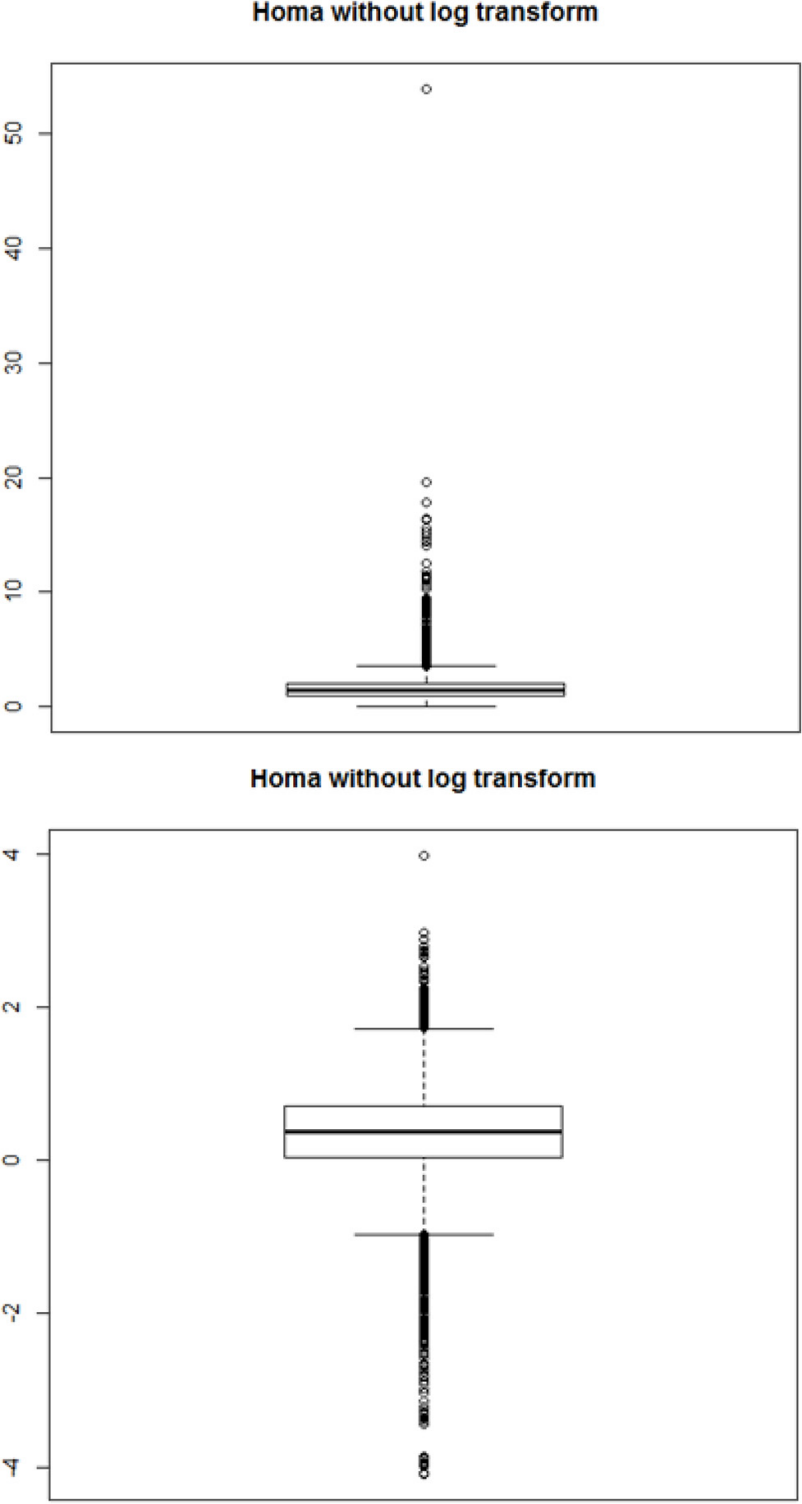
Boxplots of HOMA-IR. Before log transformation to HOMA-IR, HOMA-IR has much skewed distribution. In contrasts, after log transformation, HOMA-IR has almost symmetric distribution.

**Fig 6:**
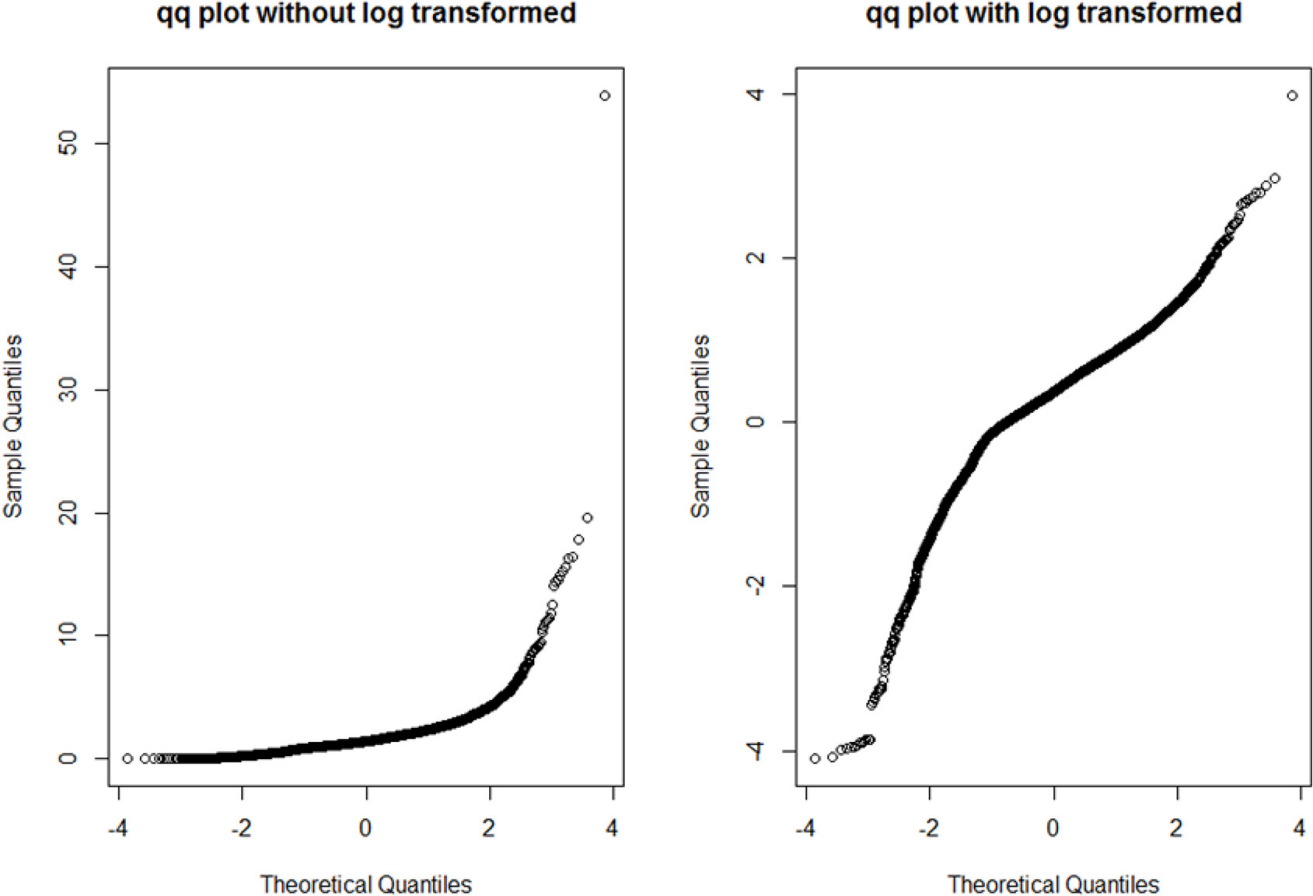
QQ-plot for HOMA-IR data. This figure shows the QQ-plots for HOMA-IR data comparing with standard normal distribution. It is found that HOMA-IR with Log-transformation acts more like samples of normal distribution in right figure.

Our proposed robust GMDR was applied to KARE data with a HOMA-IR phenotype. Fig 5 displays the boxplots of HOMA-IR without and with log-transformation. The skewed distribution of Homeostasis Model Assessment of Insulin Resistance (HOMA-IR) levels prompted many researchers to perform a log-transform before conducting the regression analysis (shown in the upper panel of Fig 5) [12]. Thus, a log-transform was also performed in our work and its boxplot is given in the lower panel of Fig 5. In spite of performing a log-transformation, the distribution of HOMA-IR levels still remained skewed, with many samples apparently qualifying as outliers. Fig 6 shows the normal quantile-quantile (QQ) plots of the HOMA-IR levels: the left panel represents the case without log-transformation and the right panel the case with log-transformation. The distribution that was obtained after the log-transformation appears to be more symmetrical than without log-transformations. Thus, our GMDR analysis was performed using log-transformed HOMA-IR levels.

Before the GMDR analysis, a single SNP analysis was first performed to reduce the computational burden on GMDR. A single SNP analysis was performed for all SNPs.

$$\log(HOMA-IR_{i})=\alpha_{0}+\alpha_{1}SEX_{i}+\alpha_{2}AGE_{i}+\gamma_{3}AREA_{i}+\alpha_{4}BMI_{i}+\alpha_{5j}SNP_{ij}+\varepsilon_{i},$$ (13)

Noteworthy is that *i* (= 1,…,8577) represents individuals; whereas, *j* (= 1,…,327,872) represents SNPs. All SNPs are ranked in the order of ascending p-values. SNPs are filtered out again by the linkage disequilibrium (LD) coefficient r^2^. Only one SNP was selected among the SNP pairs with r^2^ ≥ 0.8. Thus, the SNPs can be treated independently. After filtering SNPs via LD, we set the total number of selected SNPs as *n* / log(*n*) ≈ 585 by the sure independence screening (SIS) criterion for the purpose of performing the GMDR analysis [19].

The same model that was used for selecting SNPs was used for the GMDR analysis. Sex, age, area, and BMI were used as environmental covariates. The regression models for GMDR are given as follows:

$$\log(HOMA-IR_{i})=\beta_{0}+\beta_{1}SEX_{i}+\beta_{2}AGE_{i}+\beta_{3}AREA_{i}+\beta_{4}BMI_{i}+\varepsilon_{i},$$ (14)

The residual scores were then calculated based on this model. The threshold value of *T* was assumed as 1.5, 1.75, or 2 in the L-estimator and M-estimator. Table 3 shows the results obtained with the three GMDR methods. Each method provided a different SNP combination. These three pairs of SNPs have not previously been reported in the literature in relation to HOMA-IR. When we performed the analysis using the real data, the computational times required by the M-estimator GMDR were similar to those required for ordinary GMDR analysis. However, the L-estimator required slightly more computational time, because it contains an additional step to compute residuals.

**Table 3:** GMDR Analyses for log-transformed HOMA-IR in KARE data.

Method	Best Combination	CVC	Average Test BA
**Ordinary GMDR**	**rs576563 rs2920792**	**4**	**0.512535**
**L-estimator GMDR**	**rs4915657 rs7500315**	**9**	**0.518679**
**M-estimator GMDR**	**rs11125090 rs9353581**	**9**	**0.522633**

Table 3 shows the results of three GMDR methods. Each GMDR method provided different SNP combination.

We performed the network analysis with the top 100 SNP pairs with the highest BAs. Each node represents an SNP and each edge connecting two nodes represents an SNP pair. We defined the hub genes as genes with at least four edges. Table 4 summarizes the list of hub genes for log-transformed HOMA-IR identified by robust GMDRs and ordinary GMDR. The KCNH1 gene, which has the largest number of edges, was reported to have the function of insulin secretion [20]. Both M-estimator GMDR and L-estimator GMDR successfully identified the KCNH1 gene as a hub gene regardless of the threshold values. In addition, M-estimator GMDR with a threshold of 1.5 and L-estimator GMDR with all three threshold values identified the RYR2 gene as a hub gene. This gene is reported to be related with diabetes [21]. The PBX1 was found by M-estimator GMDR with a threshold of 2 and an ordinary GMDR. It was also reported that the PBX1 gene is related to type 2 diabetes [22]. In summary, the network analysis that was performed with the top 100 SNP pairs showed supporting evidence that the robust GMDR methods identified a larger number of hub genes that were reported in the literature than the ordinary GMDR method.

**Table 4:** Hub-genes for log-transformed HOMA-IR in KARE data with several methods.

Method	threshold = 1.5	No. nodes	threshold = 1.75	No. nodes	threshold = 2	No. nodes
L-estimator	KCNH1*	4	KCNH1*	9	KCNH1*	13
	RYR2*	7	RYR2*	5	RYR2*	9
	LAMB3	5	LAMB3	4	HPCAL4	4
	C1orf168	3	SMYD3	5	SMYD3	4
	LMX1A*	3				
M-estimator	KCNH1*	43	KCNH1*	52	KCNH1*	52
	RYR2*	6			PBX1*	4
	KIF26B	4				
	TXNRD2	3				
Ordinary GMDR	PBX1*	31
	TMEM51	5
	VSIG2	4
	KIF26B	3

Table 4 shows the hub-genes for log-transformed HOMA-IR with different threshold. KCNH1 and RYR2 are commonly detected in L-estimator and M-estimator GMDR. However, Ordinary GMDR could not detect those genes.

**Fig 7:**
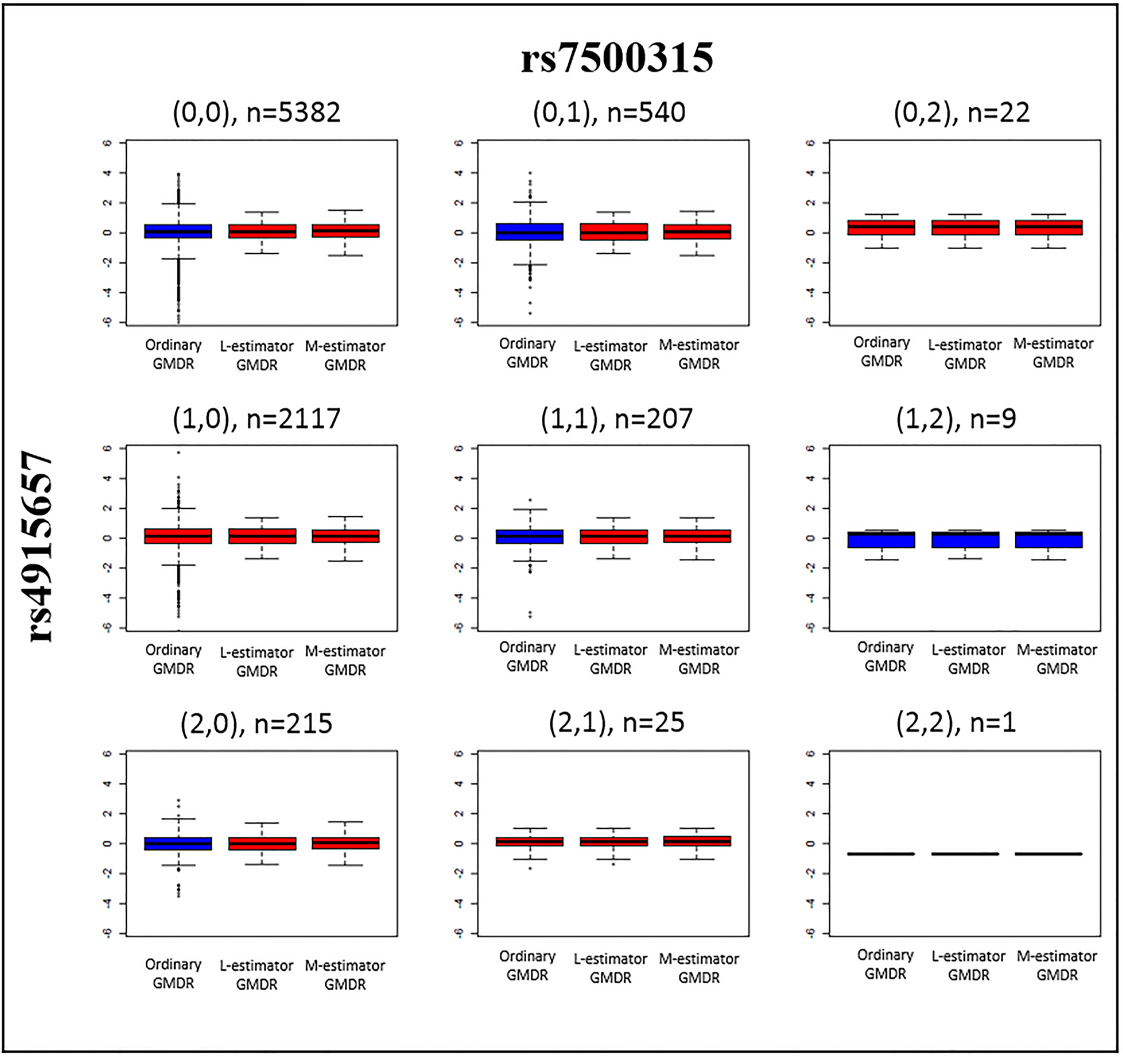
Boxplots of residuals calculated by ordinary GMDR, M-estimator GMDR and L-estimator GMDR. Each cell displays three boxplots of the GMDR methods divided by the combination of rs4915657 and rs7500316. The red and blue boxplots represents the high-risk and low-risk groups with the sum of the residuals larger than and smaller than 0, respectively.

For a selected SNP pair, we investigated the high /low risk prediction results of individual cells. Fig 7 shows the boxplots of residuals that were calculated by ordinary GMDR, M-estimator GMDR, and L-estimator GMDR, respectively. Each cell displays three boxplots of GMDR methods. The red boxplot represents the high-risk group with the sum of the residuals larger than 0, whereas the blue boxplot represents the low-risk group with the sum of the residuals smaller than 0. The high/low classification pattern of L-estimator GMDR is the same as that of M-estimator GMDR. However, the pattern of ordinary GMDR differs from that of robust GMDRs. For example, in the first cell, when the numbers of minor alleles of rs4915657 and rs7500316 are (0,0), respectively, ordinary GMDR classified this cell as a low-risk group, whereas the two robust GMDRs classified it as a high-risk group. Note that the sum of residuals for ordinary GMDR becomes negative, which is due to the negative outlying observations in the tail part. Similarly, when the numbers of minor alleles in rs4915657 and rs7500316 are (0,1), (1,1), and (2,0), the cells showed the same tendency. The different results between the robust GMDRs and ordinary GMDR could be explained by the negative outlying residuals in the tail part. Because ordinary GMDR uses the original outlying residuals as they are, the sum of the residuals is highly affected by these outlying residuals. Thus, these outlying observations can cause errors in high/low classification. This investigation demonstrates the usefulness of robust GMDRs.

## Discussion

When searching for hidden heritability, it is important to use an efficient method to establish the effects of GG interactions. To this effect, GMDR is a powerful method. However, using a simple example, it is demonstrated that the power of the GMDR method may decline in the presence of outlying observations. This problem was addressed by proposing the robust GMDR: L-estimator GMDR and M-estimator GMDR.

Simulation studies indicated that outlying observations caused the power of ordinary GMDR to decline critically. In contrast, the L-estimator and M-estimator GMDR methods were found to perform reasonably well even when outlying observations existed. When the samples are generated by assuming a Normal distribution, the L-estimator GMDR appears to outperform the other methods when the proportion of outlying observations is large. Otherwise, M-estimator GMDR and L-estimator GMDR perform similarly. If the samples are generated by assuming a Gamma distribution and there are outlying observations, L-estimator performs best. The results of the simulation study indicate that the power of GMDR with a correct distributional assumption is shown to be much larger than with an incorrect distributional assumption. Therefore, if we know the distribution of *y*_*i*_, a parametric approach, such as maximum likelihood estimation, would be recommended.

In our robust GMDR analysis, we used BA as an evaluation measure for the prediction ability. BA uses less information than continuous evaluation measures when used for quantitative traits. However, as the GMDR approach is based on transforming quantitative traits into binary high/low responses, incorporating the continuous scale information directly into the robust GMDR framework is complicated. On the other hand, QMDR does not use binary responses; instead, it uses t-statistics to measure for quantitative traits [7]. Although the GMDR and QMDR methods use different frameworks, the concept of robustness used in GMDR can be used in QMDR. Thus, in future studies, we will extend our robust scheme to t-statistics with the classical statistical method.

## Appendix

Without loss of generality, we could consider all kinds of 2^nd^ order genetic models with a normal distribution by using the following formula (15).

$$y=\sum_{j=0}^{2}\sum_{i=0}^{2}\beta_{ij}I(SNP_{1}=i,SNP_{2}=j)+\varepsilon$$ (15)

Then, we could consider the variance of y to be represented by the following equations.

$$\mathrm{var}(y)=\sigma^{2}=\sigma_{envir}^{2}+\sigma_{genetic}^{2}=E(\mathrm{var}(y|SNP_{1},SNP_{2}))+\mathrm{var}(E(y|SNP_{1},SNP_{2}))$$ (16)

$$=E(\sigma_{envir}^{2})+\mathrm{var}(\sum_{j=0}^{2}\sum_{i=0}^{2}\beta_{ij}I(SNP_{1}=i,SNP_{2}=j))$$

With this equation, we could calculate the narrow sense heritability *h* ^2^ as:

$$h^{2}=\frac{\sigma_{genetic}^{2}}{\sigma^{2}}=\frac{\mathrm{var}(\sum_{j=0}^{2}\sum_{i=0}^{2}\beta_{ij}I(SNP_{1}=i,SNP_{2}=j))}{\sigma^{2}}$$ (17)
